# Supplementary material for: Density dependence of songbird demographics in grazed sagebrush steppe
Source: PLoS One. 2023 Dec 22;18(12):e0289605. doi: 10.1371/journal.pone.0289605 (PMC10745192; doi:10.1371/journal.pone.0289605)
Supplement: S1 Table — (DOCX) [file pone.0289605.s001.docx]

**Appendix S1. List of covariates used in estimating nest density, nest survival and adult density.**

| **Name** | **Description** |
| --- | --- |
|  |  |
| ***Vegetation*** |  |
| *SGI* | grazing system category; 0 = not enrolled, 1 = enrolled in the Sage Grouse Initiative |
| *SHR* | The annual, 30m, estimated percentage ground cover of shrubs from the product available on Google Earth Engine by Allred et al. 2021 and more. See https://rangelands.app/products/ for details. |
| *GPP Mean* | The annual, 30m, estimated gross primary production from the product available on Google Earth Engine by Robinson et al. 2018. See https://zslpublications.onlinelibrary.wiley.com/doi/epdf/10.1002/rse2.74 for details. |
| *Mean LAI* | The leaf area index (LAI) as determined by 500m pixels. See https://lpdaac.usgs.gov/products/mcd15a3hv006/ for more details. |
| ***Weather*** |  |
| *Mean Precipitation* | The average daily precipitation from the growing season for that year. |
| *Max Temp* | The average daily 2-m air temperature maximum from the growing season for that year. |
| *Min Temp* | The average daily 2-m air temperature minimum from the growing season for that year. |
| ***Additional Covariates*** |  |
| *Stage* | Incubation or nestling |
| *Julian Date* | Julian Date |
| *Year* | Year of sample |
| *Species Specific Nest Density* | Species specific estimated density nests in plot based on transect distance sampling methods |
