# Supplementary material for: Density dependence of songbird demographics in grazed sagebrush steppe
Source: PLoS One. 2023 Dec 22;18(12):e0289605. doi: 10.1371/journal.pone.0289605 (PMC10745192; doi:10.1371/journal.pone.0289605)
Supplement: S2 Table — (DOCX) [file pone.0289605.s002.docx]

**Appendix S2. Comparison of biotic and abiotic covariates based on land enrollment.**

| Covariate | SGI | Non-SGI |
| --- | --- | --- |
| Mean LAI | 4.576 ± 1.283 | 4.461 ± 1.174 |
| GPP Mean | 254.274 ± 65.675 | 247.095 ± 75.001 |
| SHR | 11.292 ± 2.972 | 11.939 ± 3.771 |
| Mean Precipitation | 2.148 ± 2.35 | 2.011 ± 2.048 |
| Max Temp | 25.702 ± 2.294 | 26.087 ± 2.604 |
| Min Temp | 8.326 ± 1.57 | 8.983 ± 1.774 |
