# Supplementary material for: Density dependence of songbird demographics in grazed sagebrush steppe
Source: PLoS One. 2023 Dec 22;18(12):e0289605. doi: 10.1371/journal.pone.0289605 (PMC10745192; doi:10.1371/journal.pone.0289605)
Supplement: S3 Table — Model selection table for detection rates of Brewer’s and vesper sparrow nests using distance sampling. (DOCX) [file pone.0289605.s003.docx]

**S4 Table. Distance sampling model results for Brewer’s sparrow and vesper sparrow.**

Model selection table for detection rates of Brewer’s and vesper sparrow nests using distance sampling. Top model set is shown with different Akaike’s Information Criterion (ΔAIC_c_) ranked by descending model weight.

|  |  | Model name | Intercept | Intercept SE | Covariate estimate | Covariate SE | Detection estimate | Detection SE | AICc | ΔAIC_c_ |
| --- | --- | --- | --- | --- | --- | --- | --- | --- | --- | --- |
| Brewer's sparrow | 2016 | SHR | 3.026 | 0.499 | -0.106 | 0.039 | 0.337 | 0.058 | 142.872 | 0.000 |
|  |  | Mean LAI | 0.230 | 0.818 | 0.413 | 0.216 | 0.360 | 0.055 | 147.099 | 4.227 |
|  |  | Site | 2.064 | 0.245 | -0.003 | 0.003 | 0.376 | 0.053 | 148.270 | 0.000 |
|  |  | Mean Precipitation | 1.840 | 0.292 | -0.021 | 0.137 | 0.368 | 0.055 | 149.271 | 6.399 |
|  |  | Max Temp | 1.392 | 2.088 | 0.015 | 0.076 | 0.366 | 0.054 | 149.276 | 6.404 |
|  | 2017 | Mean Precipitation | 2.197 | 0.340 | -0.290 | 0.345 | 0.489 | 0.082 | 232.942 | 0.000 |
|  |  | Site | 2.343 | 0.339 | -0.003 | 0.004 | 0.524 | 0.077 | 233.719 | 0.776 |
|  |  | SHR | 4.136 | 1.119 | 1.156 | 0.270 | 0.466 | 0.074 | 257.636 | 24.694 |
|  |  | Mean LAI | 1.346 | 0.423 | 1.040 | 0.203 | 0.492 | 0.082 | 257.641 | 24.699 |
|  |  | Max Temp | 0.573 | 0.094 | 0.751 | 0.818 | 0.432 | 0.079 | 257.659 | 24.716 |
|  | 2018 | Mean Precipitation | 1.795 | 0.253 | 0.081 | 0.041 | 0.451 | 0.068 | 147.619 | 0.000 |
|  |  | Site | 2.006 | 0.328 | 0.000 | 0.004 | 0.480 | 0.094 | 154.215 | 6.595 |
|  |  | Mean LAI | 2.846 | 1.119 | 2.028 | 0.772 | 0.469 | 0.066 | 173.757 | 26.138 |
|  |  | Max Temp | 1.567 | 0.367 | 0.965 | 0.270 | 0.469 | 0.066 | 173.757 | 26.138 |
| Vesper sparrow | 2016 | Mean LAI | 10.716 | 3.850 | -2.588 | 0.958 | 0.051 | 0.052 | 208.065 | 0.000 |
|  |  | Mean Precipitation | 2.212 | 0.423 | -0.502 | 0.203 | 0.052 | 0.059 | 212.816 | 4.751 |
|  |  | Site | 1.702 | 0.347 | 0.005 | 0.003 | 0.469 | 0.066 | 214.191 | 6.125 |
|  |  | SHR | 2.425 | 0.867 | 1.430 | 0.772 | 0.394 | 0.203 | 245.653 | 37.588 |
|  |  | Max Temp | -2.699 | 1.785 | 1.119 | 0.440 | 0.466 | 0.074 | 245.653 | 37.588 |
|  | 2017 | Mean LAI | 4.808 | 1.734 | -0.753 | 0.440 | 0.398 | 0.079 | 191.528 | 0.000 |
|  |  | SHR | 1.006 | 0.549 | 0.093 | 0.052 | 0.442 | 0.070 | 192.731 | 1.204 |
|  |  | Site | 2.286 | 0.380 | -0.004 | 0.004 | 0.466 | 0.074 | 195.272 | 3.745 |
|  |  | Mean Precipitation | 1.995 | 0.343 | -0.105 | 0.182 | 0.438 | 0.079 | 195.839 | 4.311 |
|  |  | Max Temp | 2.321 | 1.785 | -0.018 | 0.067 | 0.432 | 0.079 | 196.563 | 5.036 |
|  | 2018 | Max Temp | 4.415 | 1.545 | -0.121 | 0.063 | 0.280 | 0.051 | 190.153 | 0.000 |
|  |  | Mean Precipitation | 1.214 | 0.303 | 0.070 | 0.050 | 0.298 | 0.048 | 191.455 | 1.302 |
|  |  | SHR | 1.850 | 0.479 | -0.019 | 0.033 | 0.327 | 0.046 | 194.529 | 4.376 |
|  |  | Site | 1.566 | 0.300 | 0.002 | 0.003 | 0.342 | 0.046 | 194.769 | 4.617 |
|  |  | Mean LAI | 1.554 | 1.288 | 0.014 | 0.205 | 0.334 | 0.047 | 194.856 | 4.703 |
