# Supplementary material for: Density dependence of songbird demographics in grazed sagebrush steppe
Source: PLoS One. 2023 Dec 22;18(12):e0289605. doi: 10.1371/journal.pone.0289605 (PMC10745192; doi:10.1371/journal.pone.0289605)
Supplement: S4 Table — Model selection table for Brewer’s and vesper sparrow nest survival. Top model set is shown with different Akaike’s Information Criterion (ΔAICc) ranked by descending model weight. (DOCX) [file pone.0289605.s004.docx]

**Appendix S4. Nest survival model results for Brewer’s and vesper sparrow.**

Model selection table for Brewer’s and vesper sparrow nest survival. Top model set is shown with different Akaike’s Information Criterion (ΔAIC_c_) ranked by descending model weight.

| Species | Model | Intercept | logLik | df | AICc | delta | weight |
| --- | --- | --- | --- | --- | --- | --- | --- |
| Brewer's Sparrow | Stage + Year + SHR + Mean Max Temp | 2.223 | -219.311 | 6 | 450.833 | 0.000 | 0.887 |
|  | Stage + Year + SHR | 2.07 | -223.094 | 5 | 456.340 | 5.507 | 0.057 |
|  | Stage + Year + Julian Date + SHR | 1.96 | -222.083 | 6 | 456.378 | 5.545 | 0.055 |
|  | Stage + Year + Julian Date | 1.82 | -228.452 | 5 | 467.055 | 16.222 | 0.000 |
|  | Stage + Year + Julian Date + Mean Min Temp | 1.84 | -228.300 | 6 | 468.812 | 17.979 | 0.000 |
|  | Stage + Year | 1.96 | -230.759 | 4 | 469.617 | 18.784 | 0.000 |
|  | Stage + Year + Precipitation Mean | 1.89 | -230.325 | 5 | 470.801 | 19.968 | 0.000 |
|  | Stage + Year + LAI Mean | 1.82 | -230.612 | 5 | 471.374 | 20.541 | 0.000 |
|  | Stage + Year + Species Specific Nest Dens | 1.81 | -230.669 | 5 | 471.488 | 20.655 | 0.000 |
|  | Stage + Year + GPP Mean | 1.91 | -230.722 | 5 | 471.596 | 20.763 | 0.000 |
|  | Stage + Year + SGI | 1.96 | -230.756 | 5 | 471.663 | 20.830 | 0.000 |
|  | Stage + Year + SGI + Species Specific  Nest Density | 1.79 | -230.660 | 6 | 473.531 | 22.698 | 0.000 |
|  | Stage | 1.25 | -234.912 | 3 | 475.883 | 25.050 | 0.000 |
|  | Year | 2.65 | -256.410 | 2 | 516.850 | 66.017 | 0.000 |
|  | NULL | 1.92 | -261.114 | 1 | 524.237 | 73.404 | 0.000 |
|  | Julian Date | 1.92 | -260.919 | 2 | 525.868 | 75.035 | 0.000 |

|  |  |  |  |  |  |  |  |
| --- | --- | --- | --- | --- | --- | --- | --- |
| Vesper Sparrow | Stage + Year + SGI | 1.19 | -350.81 | 5 | 711.734 | 0.000 | 0.714 |
|  | Stage + Year + SGI + Species Specific Nest Density | 1.23 | -350.79 | 6 | 713.738 | 2.004 | 0.261 |
|  | Stage + Year + Julian Date + SHR | 0.49 | -353.262 | 6 | 718.683 | 6.949 | 0.022 |
|  | Stage + Year + SHR | 0.73 | -356.419 | 5 | 722.950 | 11.216 | 0.002 |
|  | Stage + Year + Precipitation Mean | 1.50 | -357.202 | 5 | 724.516 | 12.782 | 0.001 |
|  | Stage + Year + SHR + Mean Max Temp | 1.36 | -356.201 | 6 | 724.560 | 12.826 | 0.001 |
|  | Stage + Year + Julian Date | 1.21 | -358.656 | 5 | 727.425 | 15.691 | 0.000 |
|  | Stage + Year + Julian Date + Mean Min Temp | 1.82 | -358.283 | 6 | 728.725 | 16.991 | 0.000 |
|  | Stage + Year + LAI Mean | 1.81 | -360.045 | 5 | 730.203 | 18.469 | 0.000 |
|  | Stage + Year | 1.477 | -361.344 | 4 | 730.764 | 19.030 | 0.000 |
|  | Stage | 1.19 | -362.603 | 3 | 731.251 | 19.517 | 0.000 |
|  | Stage + Year + Species Specific Nest Density | 1.53 | -361.281 | 5 | 732.675 | 20.941 | 0.000 |
|  | Stage + Year + GPP Mean | 1.54 | -361.286 | 5 | 732.685 | 20.951 | 0.000 |
|  | Year | 1.965 | -379.193 | 2 | 762.409 | 50.675 | 0.000 |
|  | NULL | 1.66 | -380.613 | 1 | 763.234 | 51.500 | 0.000 |
|  | Julian Date | 1.66 | -379.995 | 2 | 764.012 | 52.278 | 0.000 |
|  |  |  |  |  |  |  |  |
