# Supplementary material for: Density dependence of songbird demographics in grazed sagebrush steppe
Source: PLoS One. 2023 Dec 22;18(12):e0289605. doi: 10.1371/journal.pone.0289605 (PMC10745192; doi:10.1371/journal.pone.0289605)

**Appendix S3. Schematic of the dependent double-observer avian survey method.**

The primary (open circle) and secondary observer (dashed circle) walk single file along the transect (dotted line) within a 500 m x 500 m sampling plot. Observers survey up to 125 m on either side of the transect (dotted line). All surveys start at the lower right corner of the sample plot. Red arrows indicate the direction of travel. Figure adopted from Golding and Dreitz (2016, 2017), and Tipton et al. (2009).


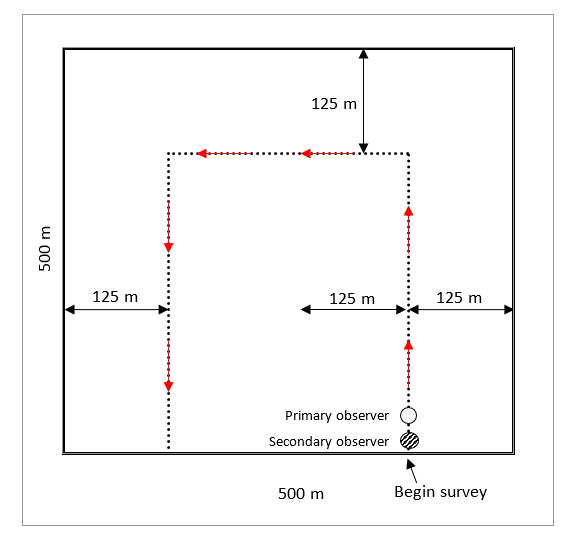

Supplement: S1 Fig — The primary (open circle) and secondary observer (dashed circle) walk single file along the transect (dotted line) within a 500 m x 500 m sampling plot. Observers survey up to 125 m on either side of the transect (dotted line). All surveys start at the lower right corner of the sample plot. Red arrows indicate the direction of travel. (DOCX) [file pone.0289605.s005.docx]
