# Supplementary material for: Density dependence of songbird demographics in grazed sagebrush steppe
Source: PLoS One. 2023 Dec 22;18(12):e0289605. doi: 10.1371/journal.pone.0289605 (PMC10745192; doi:10.1371/journal.pone.0289605)
Supplement: Attachment — Submitted filename: PlosOne Response Letter.docx [file pone.0289605.s009.docx]

Kayla Ruth

Oregon State University

Corvallis, OR, USA

(612)-270-6741

Dr. Emily Chenette

Editor-in-Chief, PLOS ONE

November 20, 2022

Dear Dr. Emily Chenette,

RE: manuscript PONE-D-22-21628 entitled “Density Dependence of Songbird Demographics in Grazed Sagebrush Steppe” by K. Ruth, L. Berkeley, K. Strickfaden, and V. Dreitz for submission as a Research Article to ‘Plos One’.

We thank the reviewers for providing helpful comments and suggestions that improved our manuscript through the rounds of revisions. We are grateful for the opportunity to resubmit the revised manuscript.

The reviews contained many useful comments and corrections. The reviewers’ main concern with the revised manuscript was whether we could address density-dependence in our study system. We believe this is a valid concern. However, this is a concern for many, and most, studies investigating if density-dependent regulation exists in populations. It is complex, and relationships are difficult to address in observational studies. But we believe it is important to consider and that the theoretical concept is neglected in songbird studies. In our resubmission, we modify text such that we are exploring the potential for density-dependence relationships and not testing if density-dependence exists within our study system.

We hope that this revised manuscript addresses all the concerns of the reviewers, and we are grateful for their thoughts and time spent reviewing the manuscript. We feel that the revised manuscript is a great improvement and will be a valuable contribution to conservation. Below we provide the reviewers’ comments in *italics* and our response below each comment in regular font.

K. Ruth is the corresponding author and is currently located at the Department of Animal and Rangeland Sciences, Oregon State University, 112 Withycombe Hall, Corvallis, OR 97331 phone: 612-270-6741, e-mail: kayla.ruth@oregonstate.edu. All authors have participated in the drafting of this manuscript and support its submission. This manuscript is being submitted for exclusive consideration by ‘Plos One’.

Sincerely,

Kayla A. Ruth

Reviewers' comments:

Reviewer's Responses to Questions

**Comments to the Author**

Reviewer #1: This manuscript compares nest and adult densities to nest survival for two songbird species in the sagebrush steppe. Given declining songbird populations and the numerous threats facing the sagebrush ecosystem, this paper represents an important contribution to the literature. However, I have some concerns, most of which may be able to be addressed through clarification and restructuring of the manuscript.

My main concern is that the focus seems somewhat confused throughout the paper. The two main points (as I understood them) were the ideas of density dependence and grazing effects. However, the paper seemed to bounce back and forth between the two ideas without really connecting them or clarifying why they should be addressed together. This makes it difficult to identify the key takeaway points. I think this could be addressed through some restructuring and further explanation, but the intro seems to focus on grazing effects while much of the rest of the paper is devoted to ideas of density dependence.

My second concern is regarding the grazing effects. I’m not convinced that you have enough information to actually test the effects of grazing. As described, you’ve only separated plots into SGI and non-SGI but there is no information on stocking rates, specific timing of grazing, duration of grazing, grazing intensity etc., in either of the systems. You state that non-SGI ranches were “generally managed less intensively”, but you also suggest that there was variation within each group (e.g., SGI was tailored to the specific ranch) and without more detail there is no reason to think that differences within a group wouldn’t be equal or greater to differences between groups. Furthermore, plots within the SGI group could be grazed at any point within the rotation and it would be reasonable to expect effects of grazing to differ if a specific plot was grazed in May vs. November (e.g., when vegetation is removed relative to the nesting period). Without any other habitat factors being considered, it is hard to isolate grazing regime as the driving factor behind any potential differences. Without more detailed information on grazing systems, it isn’t possible to judge whether this broad categorization of SGI vs. non-SGI is meaningful. Given this uncertainty, I think the emphasis on the effects of grazing, particularly in the intro, is unwarranted and overstates the reach of this paper.

*We concur with the Reviewer in discussing two very separate aspects – density-dependence and grazing –which were presented in a highly confusing manner and made the manuscript hard to follow. We have reorganized the manuscript substantially, especially in the Introduction and Discussion. Our primary interest is density-dependence, and we changed the manuscript to provide that focus.*

*In addition, we attempted to take advantage of a conservation program, the Sage Grouse Initiative, occurring in our study area. While the primary activity of this conservation program was grazing, there were many aspects of the program beyond the grazing implementation (e.g., infrastructure improvements – fences and stock tanks; private landowner engagement; economic values). In fact, during our study, we were not fully aware of when the grazing implementation occurred in the pastures of our sampling plots. We only conducted our sampling during the breeding season and suspect that some of our sample plots had grazing during seasons we were not present (e.g., fall and winter). Unfortunately, we did not collect information on grazing metrics during our study for several reasons. And we can not go back and change that at this time. Thus, we have changed the manuscript to reflect a ‘partnership-based conservation program’ and not a ‘grazing management program’ in which we explore if there is a difference in bird response based on the plot being in a pasture participating in the partnership-based conservation program.*

Specific comments
Line 31: The abstract starts with livestock grazing which, given the rest of the paper and particularly the discussion, seems to be overstating the problem, especially because not much of the discussion focuses on grazing.

*The abstract has been reworked based on reviewer comments.*

Line 44: The abstract started big focusing on evaluating the effects of grazing but, as written, this result seems tacked onto the main focus of the paper (density-dependence).

*The abstract has been reworked based on reviewer comments.*

Line 55: missing closing parentheses

*This has been fixed.*
Line 56: While I certainly don’t disagree with the statement, it is a very broad claim with no citations or explanation, and it seems added onto this paragraph as an afterthought rather than being developed as its own idea.

*The introduction was reworked based on reviewer comments.*
Line 74: Again, no citation, although I can think of several studies that have looked at grazing effects on songbirds.

*The introduction was reworked based on reviewer comments.*
Line 75: This entire paragraph doesn’t have any citations, even though the authors make some broad claims.

*The introduction was reworked based on reviewer comments.*
Line 88: This is a confusing switch from talking about density-dependence to the relationship between adult and nest densities. It isn’t clear if you’re focusing on the relationship between adult and nest densities from the angle of density dependence (more theoretically) or management (e.g., do we only care about this relationship because it could mean we could just count adults and not monitor nests), so this could be better explained and linked together.

*The introduction was reworked based on reviewer comments.*
Line 90: I think it would be helpful to clarify why we care about density dependence. The authors seem to be dancing around the issue, but it isn’t clear why, particularly from a management perspective which is how the authors have framed the paper, understanding mechanisms of density dependence is important

*We have substantially re-organized the Introduction, including providing an example, that we feel addresses the comment.*
Line 94: Here you’re bouncing back and forth between idea of grazing (started with this broadly and now have come back to it) and density dependence, which is confusing. As written, you’re just interested in how grazing affects bird densities and survival, not how it influences density dependence (which may be the case but is confusing since you had switched to the idea of density dependence).

*We recognize reviewer comments and tried to make necessary adjustments to bring together these different aspects of both ideas.*
Line 107: These seem like different objectives kind of cobbled together.

*We recognize reviewer comments and tried to make necessary adjustments to bring together these different aspects of both ideas.*
Line 109: This is the first mention of trying to compare effects of grazing and density dependence (which I think is what you’re trying to say?). That’s a curve ball to throw into your objectives.

*We recognize reviewer comments and tried to make necessary adjustments to bring together these different aspects of both ideas.*
Line 133: I’m not convinced that you have enough information on grazing regime to be able to differentiate these two or that there is a meaningful difference that would allow you to say anything about the effects of grazing. There’s no information on timing of grazing, grazing intensity or duration, or stocking rates.

*Unforunately, we were not able to aquire more specific information or grazing metrics. We recognize that these metrics would have been useful in helping tease apart grazing effects of different demographic rates.*
Line 137: What other biotic factors did you examine? Reading further, it isn’t clear that you incorporated any additional variables beyond grazing regime, year, and date. The lack of any habitat variables seems like a problem, particularly for nest survival.

*We recognize that additional biotic factors that were not collected may be influencing nest survival.*
Line 171: Do you mean 95% of birds would be observed (and not missed) using this method? As written, it is a little confusing.

*This has been edited for clarity.*
Line 182: Maybe I missed it, but did you look at effects of anything (habitat, grazing, etc) on nest density? That isn’t clear from this section. You say you used AIC to select among competing models, but don’t explain what those are.

*AIC was used to determine which key functions and adjustment terms were the best fit for the distance sampling data.*
Line 199: I assume you mean you included a random effect for ‘plot’? Not completely clear.

*This has been clarified in the section “Nest Survival Analysis”.*
Line 303: Maybe I misunderstood, but I thought you were testing the correlation between adult and nest densities. These results would suggest that the correlation was not significant for either species. If there is no correlation, how does that translate to the number of pairs being the same as the number of nests? Either this needs to be clarified or corrected.

*We concur with the reviewer about the presentation on this relationship. While the p-value suggested the relationship between nest density and adult density was not significant for either species, we believe more than a p-value needs to be considered when interpreting results from a statistical test. First, our sample size was relatively small for a correlation test. However, we felt this was the best type of statistical test to consider. Thus, why we explored the relationship between ratio of nests/adult pair and nest survival. We used the relationship also in our evaluation.*

*Lastly, we changed our text throughout the manuscript to state this is a pattern that may provide some information into the processes regulating density-dependence.*

Line 307: Given all the issues with apparent survival estimates, how is this useful?

*At the plot level, we were unable to reliably estimate nest survival estimates, due to low sample sizes. Thus, apparent nest survival was used in order to show the relationship between nest survival and nest density per adult density at the plot level.*
Line 330: Here the lack of an effect could also be because you didn’t measure or compare meaningful differences in grazing regime (i.e., that your two grazing categories are not meaningfully different).

*We have included further text acknowledging possible reasons why we had a lack of effect.*
Line 337: Any anecdotal mention of how these years differed? Any significant differences in precipitation across the 3 study years?

*Yes, this is mentioned in lines 345-348.*
Line 342: These appear to be two competing explanations, but there’s no connection/flow between them in this paragraph.

*We recognize reviewer comments and tried to make necessary adjustments to bring together these different aspects of both ideas.*
Lines 353-355: Here you start by arguing that adults may be less vigilant if other birds are around, by which I think you’re implying that they could be more successful because they don’t have to use as much of their energy reserves being vigilant? However, the next sentence talks about prey saturation being the driving factor for reduced predation. These seem to be different (although not mutually exclusive) ideas, but the writing (“In this way. . .”) seems to connect the two ideas and neither seems to be explored in enough detail.

*We recognize reviewer comments and tried to make necessary adjustments to bring together these different aspects of both ideas and to aid in clarity.*
Line 376: This is the first mention of this and seems like a concern that should be raised and explained in your methods.

*This concept has been added to the text.*
Line 392: Again, your results suggest that there was no correlation for either species.

*We concur that the relationship is not significant, however, we do see a pattern over a 3 year period that Brewer sparrow adult density is 1.5-2 times higher than nest density. We made changes to the text, especially our interpretation in the Discussion.*
Line 396: I think you could strengthen your manuscript by more (and earlier) discussion about why we care about density dependence. You seem to be arguing from a management perspective, but the links haven’t been fully developed or explained. Some additional explanation would help pull that together and help the reader understand why we should care about this paper.

*We concur with the reviewer and made changes to reflect why we care about density dependence.*

Reviewer #2: Comments to Authors, PONE-D-22-21628

General Remarks:

Note: I also reviewed a previous submission of this manuscript for a different journal. The previous and current versions of the manuscript are very similar, as are my comments and concerns. I admittedly found it disconcerting that the authors apparently elected not to incorporate most suggestions or address the concerns articulated in the previous round of reviews, as peer reviews necessitate considerable effort and time on the part of reviewers. Despite the similarities between versions, however, I re-read and evaluated the current version thoroughly.

The authors of this paper assessed relationships between livestock grazing regimes and songbird demography within a habitat type that has been altered extensively range-wide. Moreover, they attempted to assess relationships between adult density, nest density, and nest survival. I concur that understanding of grazing effects on songbirds and the extent to which density-dependence operates on nest survival (and within which contexts) is lacking. Moreover, understanding the relationship between the number of adults within an area and actual reproductive effort (herein, nest density) is important for assessing how meaningful density is as a proxy within different systems. As such, the authors have identified some important gaps in understanding, both broadly across species, and within the focal system from a conservation perspective.

I also found the writing to be generally clear and concise.

Whereas the spatial replication (n = 80, 25-ha study plots) and study duration (n = 3 years) were impressive, I still have some strong reservations about whether the study approach can suitably address the objectives related to density, and whether the system was a logical one within which to expect and evaluate density-dependence. The study took place within an area that had very low densities of the two focal species compared with other parts of their range. I also think that the authors put forth some explanations (e.g., pairs skipping breeding during some years) that are unwarranted given the approach and data, and could be unnecessarily misleading to readers in terms of furthering understanding of the species. I elaborate on these concerns, and provide some additional suggestions, below.
*We appreciate the comments of the reviewer and their assistance in improving our manuscript. We apologize if the reviewer feels we have neglected and not respected their time. However, we believe we have philosophical differences about our paper and what our study can provide in advancing our understanding of songbird populations. We hope the editor considers this difference in your assessment of our resubmission.*

Specific Comments:

With respect to the tests of grazing effects, I did not find any description of the extent of grazing pressure (e.g., average number of cattle per ha or similar). Without such information, readers cannot compare grazing levels across spatiotemporal contexts. Even providing a range of typical livestock densities present in the study plots would be helpful. Without this information, readers cannot assess whether the grazing regimes were comparatively low, moderate or high. Perhaps grazing was not influential simply because livestock densities were low enough to have minimal influence on habitat metrics that could influence food or nest predation rates for breeding songbirds, whereas at higher grazing levels negative effects could result.

*We agree with the author that to address grazing, we need some grazing metrics. We made substantial changes, given we did not have any grazing metrics to make this evaluation.*

Indeed, the introduction emphasizes habitat changes in the sagebrush steppe, and the potential influence of grazing regimes, whereas the grazing aspect seems sort of “tacked on” when it comes to the articulation of the study questions at the end of the introduction.

*We agree to this comment which is similar to reviewer 1 and have re-worked the manuscript, particularly the Introduction and Discussion.*

I also found myself craving more information about the habitat structure/composition of the SGI versus non-SGI plots. Being able to more explicitly link the two grazing regimes to potential differences in resultant habitat characteristics and then those to avian responses would be more compelling than simply comparing the effects of the two grazing treatments (especially without the inclusion of grazing intensity). This would be especially helpful for understanding why “grazing regime had a negative effect on adult pair density for Brewer’s sparrows” (lines 289 – 290).
*We agree and have made changes throughout the manuscript.*

I think more ecological context would strengthen the premise of the study. For example, the primary source of nesting mortality was nest predation. How/why did you anticipate that grazing in your system could affect egg and/or nestling predation rates (lines 95 – 96)? Do you have a sense for the primary nest predator species?
*Our paper is not about nest predation rates or cause-specific mortality of nests. Thus we do not believe adding this information is relevant to our paper. Plus, we did not specific identify nest predators in our study area, which may be several different mammals (e.g., coyotes, skunks, foxes), birds (e.g., ravens, cowbirds), and reptiles (numerous predatory snakes in the study area).*

The sample sizes of nests (N = 59 BRSP and 65 VESP) located via transect surveys were extremely low relative to other studies, especially considering the timespan (3 years) and n = 80 plots. This further exemplifies my confusion as to why the authors envisioned that their system was a logical one in which to expect density-dependent demographic responses. Certainly, local habitat quality influences the potential of an area to support individuals and the point at which density-dependence would manifest, but the authors do not describe key attributes such as mean/variation of shrub cover and height for comparison, other than to say in the Study area section that the area consists of “less shrub and forb cover and shorter shrubs than other sagebrush steppe areas”. I think at a minimum, in the discussion the authors should address their densities/nest sample sizes compared with other studies that have monitored BRSP and VESP nests, and more thoroughly address the types of systems in which density-dependent nest survival is most likely to operate.
*While we do not disagree with the reviewer's comment that our study system may not have been the most ideal, we are not sure why we should state that other systems would be more suited to address our question about density-dependent. We did the best we could, given our system. And the reality is that there have been many studies on density-dependence, especially on ungulates, that still end up stating their study system may be lacking information to look at density-dependence. Density-dependence is complex, and more samples/different densities do not make it clearer to address. Our study points out that we need to consider this form of population regulation in songbirds, a point that has seldom been stated in songbirds.*

Nest survival analysis section: I do not fully understand from the explanation provided (or supplementary data) how the two nest density covariates were calculated, with which to assess the effect of nest density on nest survival. Please clarify. Were the covariates that you used the distance to the nearest nests of each species? If so, then would this not more likely be reflective of spatial autocorrelation in rates of nest predation rather than nest densities per se?
*We did not look at distance to nearest nests in this manuscript. Thus, we are unsure of the point of this comment.*

I did not find figure 1 to be particularly necessary, as it does not convey useful spatial information beyond that described in the text.

*We concur and removed figure 1*.

I suggest standardizing your density data in figures to per square ha to be more directly comparable to other studies.

*We do not disagree with the reviewer that changing the units would be best for comparison. But the focus of our paper is not to compare it to other studies. Our focus is to explore density-dependence regulation in songbirds.*

Because ratios of the densities of adults to nests (especially for BRSP) were skewed towards adults, the authors concluded (1) that some birds may forgo breeding within some years, and (2) evidence of density-dependence. I have many reservations about these conclusions, as fleshed out in the following bullets:

• There are many reasons in my estimation for why the number of nests observed in an area should be lower than the number of adults present, as not every pair has an active nest at a given point in time. Indeed, in this study, approximately half of nests failed, often during the incubation period, meaning that at any given time, some parents within an area would be between nesting attempts. Both species also are multi-brooded, so some parents would have been between nesting attempts even after the first brood fledged.

*We do not disagree and state a few times that multi-brooding may be occurring and why we see the pattern we do.*
• Second, early-stage (build, pre-lay and lay) nests, during which adults often are not present, likely would not have been detected using the nest-searching method employed, which would bias nest numbers low. Both species will re-nest multiple times following nest failures, so early-stage nests exist throughout much of the season.

*We do not disagree with the author, but we define a nest differently. We only considered a nest as a structure with at least one egg. Thus, pre-nest (based on our definition of nest) breeding activity was not part of our study. We further believe what the reviewer refers to is another component of the reproductive effort that should be considered separately.*
• The assumption of a 1:1 sex ratio for observed adults also is potentially problematic, because detection probabilities of males (who sing) versus females (more cryptic) can be very different which could bias the number of actual pairs high.

*We do not disagree that a 1:1 sex ratio is likely not correct. It is not correct for most species. However, it’s the best we could do based on the information available. We could not locate any study that suggested a sex ratio. Further, we believe that males sign and females listen (or sing less) is not proven but highly suggested for these two songbird species.*
• The timing of relating the number of adults to nests within an area also matters. Whereas the authors indicated that surveys for adults and nest-searching were somewhat temporally matched, it was unclear at the analytical stage whether the authors compared the density of birds observed with nest densities within the same time period (e.g., week) or averaged across the season. Pairs may not have had an active nest at any given time for reasons described above. Summarizing the data across the entire season could therefore introduce considerable noise into the analyses.

*We do not disagree with the reviewer and we believe the ‘noise’ they state is an aspect the variability we estimate in our estimates of nest density and adult density.*
• The concept that BRSPs would skip breeding within a year does not make sense for a relatively short-lived bird. Certainly, some males pair later than others, and some may not pair at all, especially if they are not able to procure and defend a suitable territory. Without monitoring individually-marked birds, however, one cannot discern whether particular males or females paired or initiated a nest at any point within a season or whether individuals whose first nests failed tended to disperse within a season to try to breed elsewhere. The authors cite one paper from forty years ago to support the idea of a large proportion of male BRSP not initiating nests within a season, but those authors similarly did not mark individual birds. Suggesting that the focal species “may not breed” every year as a tactic could therefore be very misleading.

*In general, ecological theory states that long-lived species should be selective about when they breed, and short-lived species should reproduce as often as possible. It has been stated, but not quantified, that Brewer’s sparrows and vesper sparrows are short-lived (for instance, the reviewer states BRSP are short-lived). However, as the reviewer states, without individually-marking birds and following them, we do not really know if they are long-lived or short-lived. Its been assumed that they are short-lived based on their size, but there is no clear evidence. As information because available, many small birds, including songbirds, are known (there is clear evidence) to live for approximately a decade (e.g., Eastern Bluebirds, American Robins, Yellow Warblers) which is longer than that ecological theory would suggest.*
