# Supplementary material for: Density dependence of songbird demographics in grazed sagebrush steppe
Source: PLoS One. 2023 Dec 22;18(12):e0289605. doi: 10.1371/journal.pone.0289605 (PMC10745192; doi:10.1371/journal.pone.0289605)
Supplement: Attachment — Submitted filename: Response Letter.docx [file pone.0289605.s010.docx]

Kayla Ruth

Oregon State University

Corvallis, OR, USA

(612)-270-6741

Dr. Emily Chenette

Editor-in-Chief, PLOS ONE

July 2, 2023

Dear Dr. Emily Chenette,

RE: manuscript PONE-D-22-21628 entitled “Density Dependence of Songbird Demographics in Grazed Sagebrush Steppe” by K. Ruth, L. Berkeley, K. Strickfaden, and V. Dreitz for submission as a Research Article to ‘Plos One’.

We thank the reviewers for providing helpful comments and suggestions that improved our manuscript through the rounds of revisions. We are grateful for the opportunity to resubmit the revised manuscript.

The reviews contained many useful comments and corrections. The reviewers’ main concern with the revised manuscript was whether we could estimate nest density and address density-dependence in our study system. We believe this is a valid concern. However, this is a concern for many, and most, studies investigating if density-dependent regulation exists in populations. It is complex, and relationships are difficult to address in observational studies. But we believe it is important to consider and that the theoretical concept is neglected in songbird studies. In our resubmission, we modify text such that we are exploring the potential for density-dependence relationships and not testing if density-dependence exists within our study system.

We hope that this revised manuscript addresses all the concerns of the reviewers, and we are grateful for their thoughts and time spent reviewing the manuscript. We feel that the revised manuscript is a great improvement and will be a valuable contribution to conservation. Below we provide the reviewers’ comments in **bold** and our response below each comment in *italics* font.

K. Ruth is the corresponding author and is currently located at the Department of Animal and Rangeland Sciences, Oregon State University, 112 Withycombe Hall, Corvallis, OR 97331 phone: 612-270-6741, e-mail: kayla.ruth@oregonstate.edu. All authors have participated in the drafting of this manuscript and support its submission. This manuscript is being submitted for exclusive consideration by ‘Plos One’.

Sincerely,

Kayla A. Ruth

Reviewers' comments:

Reviewer's Responses to Questions

**Comments to the Author**

**Reviewer #2: The authors have softened the focus on grazing effects throughout the paper which was an improvement and is logical given that information on grazing intensity was not available.

The new focus on the SGI, however, seems a bit undeveloped. For example, the authors state in the introduction (lines 85 – 86) that: “However, this conservation program may influence density dependent responses in sagebrush steppe songbirds.” How? Why? What specifically about the implementation of the SGI and how it tends to affect sagebrush habitats make it potentially influential on density dependence per se?**

*We adjusted the phrasing and re-worded our reasons for testing SGI implementation.*

**Moreover, without a better understanding of how SGI versus non-SGI study plots differed in ways pertinent to nesting songbirds, such as resultant habitat structure or composition, I am not convinced that the comparison is particularly useful for practitioners.**

*We included additional vegetation metrics to quantify SGI vs non-SGI study plots and included these covariates in our analyses when relevant.*

**The authors also state in the abstract that they “found evidence for density dependence when assessing the ratio of adult pair density to nest density.” How? In what way? Demonstrating that adult birds were detected more frequently than nests within a particular time period does not necessarily suggest that density-dependence was operating. Moreover, birds were not individually marked so how were pairs definitively delineated?**

*We concur with the reviewer and have rewritten the abstract.*

**First paragraph in the introduction: Logical flow could be improved, as the scope of inference jumps around a bit. For example, the authors begin with sagebrush songbirds and then zoom back out to discuss density dependence in general. I suggest beginning with the bigger-picture context then leading up to the specific system and questions.**

*The first paragraph has been rewritten for improved logical flow.*  **The following sentence in the introduction: “Density dependence has a paucity of information regarding how it affects vital rates in avian populations” does not make sense as written.**

*This sentence has been reworded.*  **Lines 91 – 92: What was the biological rationale for your a priori hypothesis that nest survival for sagebrush songbirds is density-dependent? I still don’t understand why with such low densities of a species one would expect density-dependent responses. Two BRSP nests within a 25-ha plot, for example, is very sparse.**

*We have added text to highlight the lack of understanding of density dependence in any population size and density level. Additionally, an alternative hypothesis of no relationship has been added.*  **I still do not agree with the assertion, moreover, that two adult birds should necessarily equate to one nest within a set timeframe an, for many reasons that I articulated at length in my previous reviews. This does not negate, however, the potential importance of evaluating how the density of adults (as typically assayed by monitoring programs etc.) generally relates to nesting densities within an area.**

*While we agree that we may have sometimes counted two birds of the same sex as a nesting pair, we do not agree that, for instance, we should always count a singing bird as a male and a silent bird as a female. We have noted instances of female birds singing songs in other studies we have conducted with banded birds. Moreover, how do we then count birds giving chip calls? No matter how we decide to delineate nesting pairs, we would always be making some assumption about the sex of the bird based on potentially spurious cues. Therefore, we opted for the option that was most straightforward to implement across all surveys.*

**Still unclear to me is the temporal period across which nest densities were compared with adult densities. Were the data summarized within a plot and year? If so, did you relate the average or maximum number of adults and nests detected within a plot and year to one another?**

*In the methods section we added text for clarity as to how nest and adult densities were calculated. They were calculated at the plot level for each year. Those values were then used to look at the relationship between nest density and adult density at the year-specific plot level.*  **Do you think the detection probabilities of nests (0.38) and adults (0.48) was sufficient (and sufficiently similar) to justify comparison?**

*We do feel the estimates of detection were sufficient to make the comparison. Detection is incorporated into the estimates of nest density and adult density, and their corresponding levels of precision. Thus, we believe our estimates of nest density and adult density are more accurate than not accounting for detection.*

**Line 228: what is meant by “nesting territories?”**

*This has been reworded for clarity.*  **Lines 306 – 314: I think referring to density-dependent population regulation is outside the scope of the study. Many factors can influence population growth, and some vital rates have much higher sensitivity to actual lambda than others.**

*Due to the new analysis results, this paragraph was removed.*

**Lines 307 to 314: The first part of this text is written in the form of predictions which better belongs at the end of the introduction when questions/hypotheses/predictions are presented. The last sentence is interpretation which typically belongs in the discussion section.**

*Due to the new analysis results, this paragraph was removed.*

**Discussion, first sentence: Again, why (in this system) did you hypothesize that nest density would be density-dependent? Nest densities were very low, both compared to other sagebrush steppe and other habitat types. Without more biological explanation the focus is not convincing.**

*We added context and biological explanation.*  **Lines 327 – 328: Just because nest densities were positively correlated with nest survival does not mean it had anything to do with density, per se. Alternatively, more birds may have been attracted to settle in areas of higher habitat quality.**

*We are unclear by the reviewer’s point, but we will try to address. In the manuscript, we state “Higher quality habitat sites are associated with higher vital rates, including survival and reproduction (Rodenhouse et al. 1997)”. Thus, if more birds are attracted to higher habitat quality for nesting, then more nests and higher nest success. Its when high quality sites are filled, and individuals forgo nesting or use suboptimal habitat, nest density and nest success will decrease.*

**Lines 336 – 338: I remain steadfast in my opposition to the conclusion that just because more adult birds were detected than nests suggests that some birds may not have initiated a nest within a given season. There just simply is not enough evidence to conclude this with the relatively low detection probabilities of nests and adults, and the inability to differentiate among individuals, in addition to all of the other arguments I suggested in my last reviews of this paper. This also is not a necessary conclusion to include in terms of the primary objectives.**

*We acknowledge some of the reviewer’s concerns. We re-organized and re-worded our text to soften the language we had earlier. However, we are not the only study suggesting that not every Brewer’s sparrow breeds every year. We include a reference (Reynolds 1981) in the manuscript. Further, we are unclear on the logic of low detection probabilities as a reason there is not some evidence. We estimated detection (and its precision) and accounted for detection in our estimates of nest density and adult density and their corresponding confidence intervals. Our detection probabilities could have been lower than they were, but we could still have the same level of precision in our estimates. Similarly, detection could have been rather high (e.g., 0.90), and we still could have the same level of precision in our estimates suggesting that adult density was higher than nest density. Thus, we are unclear of the reviewer's point of ‘relatively low detection probabilities’ is a reason we can NOT suggest there are more adults than nests for Brewer’s sparrows in our study area.*

**Lines 383 to 383: Most arid shrubland and grassland songbird nestlings do not elicit begging calls, which parallels their general propensity towards crypticity.**

*This section of the discussion was removed. However, observers in the field occasionally heard begging calls when approaching nests monitored in this study.*

**Reviewer #3: General comments:
Although I did not review the original submission, from the track changes and response to reviewer comments, I found the manuscript revision to be greatly improved from the original submission. However, I do not feel that the authors fully addressed all of the original reviewer comments and I would like to see those better addressed prior to publication. I do feel that the authors provided the caveats appropriately that the original Reviewer 2 requested, although they could be wordsmithed for clarity. I have listed the specific original comments still needing to be addressed below. These are followed by specific comments from myself. I found the article on the whole to be well written and concise, but I have three major concerns. The first is the use of apparent nest success. I agree with the original Reviewer 1 that it is not useful, and I would go further and caution that it could be harmful/misleading. The authors' response that their sample size was too low to use other methods was unsatisfactory to me. I would like the authors to use a more reliable estimator of nest success. It does not have to be logistic exposure, but it should not be apparent nest success. My second concern is that the results and discussion for the nest survival section do not match the table S3. I am unsure if this is a typo in the table or if something else is going on, but as presented the results and discussion are not supported by the modelling results. This is specific for Vesper's sparrow where the authors say the top model includes nest densities from both species but list the top model as Stage + Year + SGI with no mention of density. Lastly, I have some concerns regarding their methods that need clarifying, including how accurate their distances were that were included in the distance sampling, and the appropriateness of excluding auditory detections from their surveys, which is in contradiction to the papers they cite. I do believe that their final conclusion that adult pair densities are not a good proxy for species such as Brewer's sparrows, has management implications and would contribute greatly to the literature.

Original reviewer comments not fully addressed:

Line 137: What other biotic factors did you examine? Reading further, it isn’t clear that you incorporated any additional variables beyond grazing regime, year, and date. The lack of any habitat variables seems like a problem, particularly for nest survival. We recognize that additional biotic factors that were not collected may be influencing nest survival.

This was not addressed in the revised manuscript. The authors state the tested "biotic factors" but did not list them I the text.**

**Furthermore, Reviewer 2's comments below were not fully addressed:**

*We have included further biotic and abiotic factors as well as tables in the appendix to explain those covariates.*

**"Nest survival analysis section: I do not fully understand from the explanation provided (or supplementary data) how the two nest density covariates were calculated, with which to assess the effect of nest density on nest survival. Please clarify. Were the covariates that you used the distance to the nearest nests of each species? If so, then would this not more likely be reflective of spatial autocorrelation in rates of nest predation rather than nest densities per se?
We did not look at distance to nearest nests in this manuscript. Thus, we are unsure of the point of this comment." and "The sample sizes of nests (N = 59 BRSP and 65 VESP) located via transect surveys were extremely low relative to other studies, especially considering the timespan (3 years) and n = 80 plots. This further exemplifies my confusion as to why the authors envisioned that their system
was a logical one in which to expect density-dependent demographic responses. Certainly, local habitat quality influences the potential of an area to support individuals and the point at which density-dependence would manifest, but the authors do not describe key attributes such as mean/variation of shrub cover and height for comparison, other than to say in the Study area section that the area consists of “less shrub and forb cover and shorter shrubs than other sagebrush steppe areas”. I think at a minimum, in the discussion the authors should address their densities/nest sample sizes compared with other studies that have monitored BRSP and VESP nests, and more thoroughly address the types of systems in which density-dependent nest survival is most likely to operate."

I would like a table in the supplementary material listing variables they tested and their definitions or more clarity as they were calculated. I concur with reviewer 2 that there is a lack of explanation as to how the nest density covariates were calculated. The authors only state in a footnote "
3Interspecific Density = combined nest density of Brewer’s and vesper sparrow nests 4Intraspecific Density = nest density of respective species" I would like to see a better explanation of the calculations and how this was included in the analysis for the density dependence.**

*This table was edited for clarity and an additional supplemental table was added explaining the variables used.*  **Line 307: reviewer 1 questioned the use of apparent nest survival. I concur with the problem (also see my comment for lines 261-264 in the new manuscript.) The author's response that the sample size was too low is not a good enough reason to include apparent nest success in a manuscript when we know how flawed it is. Unsuccessful nests are less likely to be found than successful. I would like the authors to at least use the Mayfield estimator or a different method if the logistic exposure method does not have the sample size at the plot level.**

*We have removed the apparent nest survival figure and used only estimated nest success estimates.*

Specific comments:
**Line 110: authors switch from past tense to present tense
Line 113: duplicate comma
Line 118: missing the word "Were" …. "Of the 80 plots, 40 [were] privately managed…"**

*These small typos were corrected.*

**Lines 126-127: Authors state they examined other "biotic and abiotic factors…" please list them, or reference a table. I only see stage, year, SG, day of year and density referenced in the appendix. What about vegetation variables? It appears the authors did not collect vegetation data, but GIS level data would give some ideas of differences across sites, especially for sagebrush cover. I suspect that differences in Brewer's sparrow numbers have more to do with the shrub cover than they do with density dependence. In lines 111-112 they mention there is less shrub cover, but I would like to see some sort of summary. As a sagebrush-obligate species, not including any measure of sagebrush cover seems very problematic when assessing density dependence, as the number of nests/individuals that the area could support is likely related to the shrub and forb cover among other factors.**

*We included additional biotic and abiotic factors. As the reviewer suggests, we found shrub cover to be predictive on nest success. We also included supplemental tables explaining the covariates used more clearly.*

**Line 131: how big are the "plots?" 500 m long at least I assume… but there is no mention of how many transects were placed parallel 100 m across, or how wide the plots are. Would be helpful to get a feel for how well the plots were surveyed, as the nest densities are rather low.**

*We added text to more clearly define the size of the plots and how the plots were surveyed.*

**Line 140: The authors indicate they used GPS coordinates to measure the distance between the transect line and nests. How far on average was this distance and what is the accuracy of the GPS used? If observers were walking 5m from the transect line, I imagine that might be the average, and that coincidentally is the accuracy of many GPS units. Why did the observers not carry measuring tapes and measure the distance? If the distances from the transect were typically much greater than the accuracy of the GPS then I can buy this method, but for a distance sampling analysis I find it concerning if I cannot trust the accuracy of the distances measured.**

*We added text and references regarding GPS accuracy and the average distance from the transect line that nests were found.*

**Line 141: What was the range of dates these surveys started? A Day of year range- which the authors call Julian date would be helpful if surveys changed based on year.**

*A date range was added in the text.*

**Line 147: abandonment usually occurs earlier than brood rearing unless the adult is killed, how did you determine abandonment during the incubation stage? Time to estimated hatch? Several visits without observing an adult?**

*We added text referring to how we waited until after anticipated hatch date to determine abandonment.*

**Lines 156-157: immediately before nest surveys: this read to me as the same day, but the next sentence implies it was the next, consecutive day, and the latter half of line 157 has a range of 1-3 days. These sentences are confusing. Maybe list how often percentage-wise they were conducted "immediately before" and better define what that means. Same day? Different days?**

*The text has been edited for clarity.*

**Line 159: The authors indicate they use the double observer method developed by Nicholas et al but indicate they only recorded visual detections (line 168) of the songbird species studied. This is not actually the method described by Nicholas et al, as in their methods they identify all seen and heard: "The primary observer identifies all birds seen and heard and communicates (via speech and gesture) to the secondary observer the species detected and the direction and general distance of the detection." Why did the authors only use visual identification of species when their cited source uses auditory? This strikes me as potentially problematic considering the cryptic nature of these songbirds. This difference in method may have affected the density calculations used in density dependence variables. The citation #23 also included auditory detections in their analyses.**

*The citations have been reviewed and fixed. Auditory observations were often used to confirm the identify of the bird visually observed. We also note that Golding and Dreitz 2016 only used visual detections in their analyses. Further, while citation #23 included auditory detections, it was 7% of all observations (119 total observations) which likely would not significantly change that studies results.*

**Lines 160-161: authors reference 2 papers by the same author in the same system, but one is an occupancy paper, which is a different analysis than the authors here use, the second did indicate they included auditory detections (see my comment above). Furthermore, these papers are for burrowing owls and plovers, which I assume have a rather different type of detection. Burrowing owls are less vocal than sagebrush obligate songbirds and more easily spotted visually. Has this method ever been used for songbirds?**

*We had reviewed and fixed citations to support our methods. Additionally, a new paper using this method was added.*

**Line 163: this citation is not correct for this statement as it is not the original and refers to "(Scott and others 1981)" It is also discussing the 125m distance in a different context- establishing the minimum distance between surveys. The cited reference recommends a larger distance in open landscapes. It seems the authors may have double counted adults per language from their cited reference: "In addition, the maximum detection of virtually all individuals of most species is less than 250 m (Wolf and others, in this volume). In open environments, this minimum distance should be increased due to the greater detectability of birds. " I would like the authors to better explain why that distance was chosen- do they think they double counted birds? Why the U shape instead of a straight line that would prevent duplication?**

*We included additional citations for why this distance was chosen. We train observers on the project to keep track of birds to avoid double counting, including techniques suggested by Nichols et al. (2000).*

**Lines 178-179: why were these the candidate models included? Citation 28 is a nest survival analysis and does not make sense to cite here.**

*This was edited for clarity.*

**Lines 180-181: I don't see a table 1, which is referenced here in the text**

*The tables have been reorganized and correctly referenced.*

**193-195: worded a bit confusingly- was year a random effect or just plot?**

*This was edited for clarity.*

**Lines 196-198: I would like more details on how the nest density interspecific and intraspecific variables were calculated. Were these per site? Authors mention per year, but summarized across what scale? I need more information.**

*We have simplified the nest density metric to only be for species-specific nest density and text has been added to explain how those values were used. Nest density was calculated at the year-specific plot level.*

**Lines 200-202: were there other species' nests present that could be contributing to interspecific density dependence? Sage thrasher, sagebrush sparrow, etc? Do the authors feel this value fully encompassed interspecific density dependence?**

*There were very few observations (< 1%) of other sagebrush-steppe songbirds in our study. Our study area is on the sagebrush/grassland fringe in which sagebrush shrubs are most often > 0.5 m. Further, we re-did the analysis and removed text regarding interspecific nest density.*

**Line 204: interactions implied multiplicative interactions to me, can you specify if the only interactions tested were additive combinations? Or did you test multiplicative and not show them in the table?**

*Text and the supplementary table have been edited for clarity. Only additive models were used.*

**Line 210: S3 table is for nest survival- what is the author trying to reference here? Is there a missing table for adult density?**

*The tables have been reorganized and correctly referenced.*

**Lines 227-228: given these violations of that assumption, do the authors still feel the results are valid or useful? During the adult surveys was the behavior of the bird noted? If so this might give some indication of sex (perched and singing versus silent or only producing call notes). As written the reader is left wondering if the results are useful. Perhaps language as to how these violations would effect results (artificially increase or decrease) could be helpful.**

*We have edited the text to be more clear. While we did not note behavioral evidence of individual songbird sex, we do not agree that, for instance, we should always count a singing bird as a male and a silent bird as a female. We have noted instances of female birds singing songs in other studies we have conducted with banded birds. We would always be making some assumption about the sex of the bird based on potentially spurious cues. Therefore, we opted for the option that was most straightforward to implement across all surveys.*

**Lines 248-249: lack of a difference does not mean Vesper's sparrow failed to respond to the SGI program, but it could be due to low sample size. 65 nests across 80 plots over 3 years is relatively low nest density.**

*We added text to recognize our small sample size used for nest density estimates.*

**Line 251: perhaps conservation regime instead of grazing regime? To be more consistent with your manuscript? It would also be really helpful if you indicated if the CIs do or don't overlap somehow, like with an * Just curious- but why did you summarize across the SGI and non-SGI but not across years? Seems like that would be the more interesting comparison (SGI vs non-SGI summarized across 2016-2018)**

*This table has been fixed using the reviewer’s suggestions.*

**Line 260: oh wow, your nest numbers are not as low as I thought- seems like you found a lot of nests opportunistically. Why do you think that is? Were these missed in your plots during the nest searching or were they outside of the search area? Over half of your Vesper nests were found opportunistically, that is surprising to me. This might be helpful to mention with a (n= XX) in the results following your words opportunistically and distance sampling.**

*The nest sample size used in distance sampling could only include nests found on nest searches and only in plots with more than two nests found, which therefore led to more nests that were not used in the distance sampling analysis to be used in the nest survival analysis. Text has been added to clarify this.*

**Lines 261-264: seems like you shouldn't use apparent nest success here. Why not use your modelled data to provide an estimate of overall nest success or at least Mayfield's. Apparent nest success is often more misleading than helpful. I have never seen nest survival lumped across species, especially ones quite different. This seems odd for lines 263 and 264. Predation rates would be more informative by species, not lumped together.**

*The text has been edited for clarity and more specific breakdowns by species.*  **Lines 270-271: this statement is not supported by your AIC table. Table S3 does not indicate that density is in the top model. You also mention total nest density here and define it as nest density for both species. You define interspecific density that way in your table, but that is shown in the second model, not the top model.**

*The tables have been reorganized and correctly referenced.*

**Line 285: ah, this is where you say the size of the plot. Please include this in the methods section.**

*This has been included in the methods section.*

**Line 307: See above comment- I agree with reviewer #1 and do not feel this was addressed in this revision**

*This was addressed in this revision.*

**Line 326: Perhaps there is an error in the S3 table? As this statement is not supported by your modelling results where the top model is listed as Stage + Year + SGI. The top model is also competitive with two others, which deserves discussion. I would need to see a supplementary table with the parameter estimates of the top model (or models if several are within 2AIC) to be sure of the results and interpretation the authors are making**

*The tables have been reorganized and correctly referenced.*

**Lines 328-329: inverse density dependence is an odd way of putting this. It appears that many individuals are selecting areas of high quality habitat that are able to support more nests, and those who chose these areas are rewarded with higher success rates. I assume if the authors had any type of habitat quality data they could dig into what is going on here to promote higher nest survival in such areas. To describe it as inverse density dependence suggests the Allee effect where populations at low numbers increase in growth rate/ nest survival rate- which is not the case here. I suggest re-wording this sentence.**

*Due to the corrected analysis, we removed text regarding inverse density dependence.*

**Lines 330-331: there needs to be a bit more to finish this thought- see reviewer #1 comment from lines 353-355. I agree with the reviewer that you seem to be implying "that adults may be less vigilant if other birds
are around, by which I think you’re implying that they could be more successful because they don’t have to use as much of their energy reserves being vigilant?" I think you only addressed the second portion of their original comment and changed the next sentence to say additionally instead of in this way, but you didn't finish your thought.**

*Due to the corrected analysis, we removed text regarding this.*

**Lines 333-335: see previous comment about your interspecific density metric**

*The interspecific density metric has been reworked and clarified.*

**Lines 344-349: I see this was added to address Reviewer 2's comments. I think this does that, but could be re-worded for clarity using what the hypothesis is rather than referring to rejecting your hypothesis and having the reader hunt back through the text for what the hypothesis was exactly.**

*We added text to clarify what hypothesis was rejected and why.*

**Lines 350-353: I think I understand what you mean, but consider re-wording for clarity, as to what the considerations should be- just that managers know there are birds that are non-breeders or migrants? And not assume the population is doing as well? What's the implication?**

*This has been reworded for clarity.*

**Lines 358-359: consider re-wording "The exact mechanisms that caused the differences for Brewer's sparrows" reads odd. Which differences? In nest density?**

*This has been reworded for clarity.*

**Line 360-362: perhaps you are missing a word here? I think you are trying to say that Nest density and nest success did not differ for the thick billed longspur**

*This has been reworded for clarity.*

**Line 362: similarly**

*This has been reworded for clarity.*

**Lines 360-363: Maybe a sentence or a few words connecting these thoughts that other species in the area were not affected by SGI status.**

*We added text to connect these thoughts and reference similar studies in the area regarding greater sage-grouse.*

**Lines 395-397: I'm not sure you need this statement. Now that the paper is less focused on grazing it seems a bit out of context.**

*This line was removed as suggested by the reviewer.*
